# Supplementary material for: Non-medical determinants of perinatal health: protocol for a systematic review with meta-analysis
Source: BMJ Open. 2019 Sep 27;9(9):e031437. doi: 10.1136/bmjopen-2019-031437 (PMC6773282; doi:10.1136/bmjopen-2019-031437)
Supplement: Author's manuscript [file bmjopen-2019-031437.draft_revisions.pdf]

BMJ Open

BMJ Open is committed to open peer review. As part of this commitment we make the peer review history of every article we publish publicly available.

When an article is published we post the peer reviewers' comments and the authors' responses online. We also post the versions of the paper that were used during peer review. These are the versions that the peer review comments apply to.

The versions of the paper that follow are the versions that were submitted during the peer review process. They are not the versions of record or the final published versions. They should not be cited or distributed as the published version of this manuscript.

BMJ Open is an open access journal and the full, final, typeset and author-corrected version of record of the manuscript is available on our site with no access controls, subscription charges or pay-per-view fees (http://bmjopen.bmj.com).

If you have any questions on BMJ Open's open peer review process please email info.bmjopen@bmj.com

BMJ Open

Non-medical determinants of perinatal health: protocol for a systematic review with meta-analysis

Journal:	*BMJ Open*
Manuscript ID	bmjopen-2019-031437
Article Type:	Protocol
Date Submitted by the Author:	03-May-2019

Complete List of Authors:	Daalderop, Leonie; Erasmus MC, Obstetrics and Gynaecology<br>de Groot, Marjolein; Erasmus MC, Obstetrics and Gyneaecology<br>van der Meer, Lindsey; Erasmus MC, Obstetrics and Gynaecology<br>Steegers, Eric; Erasmus MC,<br>Bertens, Loes; Erasmus MC, Department of Obstetrics and Gynaecology

Keywords:	Perinatal health, social determinants of health, systematic review, meta-analysis, non-medical determinants

SCHOLARONE™
Manuscripts

Non-medical determinants of perinatal health: protocol for a systematic review with meta-analysis

Leonie A Daalderop[1 2], Marjolein W de Groot[1], Lindsey van der Meer[1], Eric A P Steegers[1], Loes C M Bertens[1]

[1]Department of Obstetrics and Gynaecology, Erasmus MC, University Medical Centre Rotterdam, PO Box 2040, 3000 CA, Rotterdam, The Netherlands

[2]Dutch Research Institute for Transitions, Erasmus University Rotterdam, Postbus 1738, 3000 DR Rotterdam, The Netherlands

Corresponding author: Leonie A. Daalderop

Address: Erasmus University Medical Centre, Department of Obstetrics and Gynaecology, PO Box 2040, 3000 CA, Rotterdam, The Netherlands

Telephone number: +31 (0)10 703 15 45

E-mail address: l.daalderop@erasmusmc.nl

Word count: 1,209 words.

Key words: Perinatal health, non-medical determinants, social determinants of health, systematic review, meta-analysis

For peer review only

ABSTRACT

Introduction: Research focusing on the associations between non-medical determinants and unfavourable perinatal health outcomes is increasing. Despite increasing knowledge on this theme, it still remains unclear to what extent social, environmental, and lifestyle factors contribute to these unfavourable outcomes. Therefore, we aim to provide a systematic review, preferably with meta-analysis, in order to provide insight into the associations between non-medical determinants and perinatal mortality, preterm birth, and small for gestational age (SGA).

Methods and analysis: Observational studies performed in European countries, studying the associations between non-medical determinants and unfavourable perinatal health outcomes will be included. Primary outcomes of interest are: perinatal mortality, preterm birth, and SGA. To retrieve potential eligible articles, a systematic literature search will be performed in the online databases MEDLINE (PubMed), EMBASE, Web of Science, Cochrane, and Google Scholar. Additionally, a reference list and citation search will be performed. Data of the included articles will be extracted using a standardised and piloted data-extraction form. Risk of bias will be assessed using the Newcastle-Ottawa Scale. The study selection and data extraction process will be performed by two reviewers independently. Disagreements will be resolved through discussion with a third reviewer. The pooled effects will be calculated separately for each association found between one of the outcome measures and the non-medical determinants, using a random effects model. Heterogeneity of the studies will be assessed using the I^2 statistic. In addition to this, the population-attributable fraction (PAF) will be calculated based on the effect estimates of the meta-analysis. The PAF will be used to estimate the contribution of each non-medical determinant to the burden of every single outcome measure within the European context.

Ethics and dissemination: No ethical approval is necessary for a systematic review with meta-analysis. The findings will be published in a peer-reviewed journal.

Prospero registration number: CRD42018056105

ARTICLE SUMMARY

Strengths and limitations of this study

- This systematic review with meta-analysis will provide a comprehensive overview of the existing literature on the associations between non-medical determinants and perinatal mortality, preterm birth, and SGA.
- The results of the meta-analysis together with the population-attributable fraction (PAF) can highlight the relevance of the studied non-medical determinants as well as the need to develop preventive strategies aimed at diminishing unfavourable perinatal health outcomes.
- This systematic review focuses on studies performed in European countries with more or less comparable health care systems, which limits the generalisability of our findings to countries with different health care systems or a different welfare state.

For peer review only

INTRODUCTION

Preterm birth and being small for gestational age (SGA) affect 11 to 27 percent of all live births worldwide and are the leading cause of perinatal mortality.[1-3] Beside the negative impact of well-known medical and obstetric risk factors, risk accumulation of non-medical determinants (e.g. social, environmental, and lifestyle factors) can explain additional variation in the occurrence of unfavourable perinatal health outcomes (i.e. perinatal mortality, preterm birth, and SGA).[4-9] In high-income countries, the risk of unfavourable perinatal health outcomes is highest among women living in socioeconomically deprived areas.[4 7 10 11] The negative impact of social deprivation on perinatal health outcomes extends into adulthood, with long-term health consequences for the affected children and therewith major implications for public health.[12]

Research focusing on the associations between non-medical determinants and perinatal health outcomes is increasing. Several studies reported non-medical determinants as risk factors for unfavourable perinatal health outcomes.[4 6 7 10 11 13 14] Additionally, studies indicate that the negative impact of non-medical determinants is often the result of clustering of two or more risk factors.[8 9] However, many other studies do not show associations between non-medical determinants and unfavourable perinatal health outcomes.[15-19] Furthermore, research on this topic often focuses on the associations between a limited number of non-medical determinants and a single outcome measure, such as preterm delivery, low birth weight, or SGA. Given the global burden of both non-medical determinants and unfavourable perinatal health outcomes, a comprehensive overview of relevant non-medical determinants and the way they influence each other and therewith perinatal health outcomes is necessary to deliver the best possible care for vulnerable pregnant women.[3 20 21]

Therefore, we aim to provide a systematic review, preferably with meta-analysis, in order to investigate the impact of a broad range of non-medical determinants on unfavourable perinatal health outcomes. This can help scientists as well as healthcare providers to thoroughly understand the role of non-medical determinants with regard to unfavourable perinatal health outcomes.

For peer review only

METHODS AND ANALYSIS

The research methods follow the PRISMA-P (Preferred Reporting Items for Systematic Reviews and Meta-analyses Protocols) guidelines (supplementary file 1).[22] This systematic review with meta-analysis is registered with the PROSPERO prospective register of systematic reviews on November 12, 2018 (CRD42018056105).

Eligibility criteria

Studies will be selected according to the criteria outlined below.

Study design and participants

We will include all observational studies (i.e. cohort, case-control, and cross-sectional studies) reporting on pregnant women that study the associations between exposure to non-medical determinants during pregnancy and unfavourable perinatal health outcomes (i.e. fetal complications during pregnancy, labour, and/or delivery). Exposures can be measured at the individual or neighbourhood level, or both. Studies performed in European countries are eligible for inclusion, as these are countries with a more homogeneous population and, more or less, comparable health care systems. Experimental studies, case reports, editorials, commentary, and clinical guidelines will be excluded. Additionally, systematic reviews and meta-analyses will be excluded; however their reference lists will be screened for potential eligible articles.

Determinants of interest

This systematic review will focus on non-medical determinants that may influence perinatal health outcomes. Determinants that will be taken into account are:

- Socioeconomic status (SES);
- Neighbourhood deprivation;
- Marital status;
- Housing;
- Domestic violence;
- Employment status;
- Educational level;
- Insurance status.

Outcomes

The primary outcomes are:

- Perinatal mortality: death occurring between 22 weeks of gestational age and 7 days after birth;
- Preterm birth: delivery of a live-born baby before 37 completed weeks of gestation;
- SGA: birth weight below the 10th centile adjusted for ethnicity, parity, gestational age, and gender.

Secondary outcomes are:

- Neonatal mortality: death of a baby occurring within the first 28 days of life;
- Low birth weight: birth weight below 2,500 grams.

Information sources and search strategy

The following electronic online databases will be searched: MEDLINE (PubMed), EMBASE, Web of Science, Cochrane, and Google Scholar. A search strategy is developed for EMBASE (table 1), using Emtree terms and free text terms related to non-medical determinants, perinatal health outcomes, pregnant women, and the study design. The search strategy will be amended for use in other databases. The database searches will be supplemented by screening reference lists and performing

For peer review only

a citation search of included articles. Only articles written in English will be included. No limitations will be applied regarding publication date.

Table 1. Search strategy in EMBASE

Element	Search items
Non-medical determinants	('social determinants of health'/exp OR 'marriage'/de OR divorce/de OR 'female by marital status'/exp OR 'educational status'/exp OR 'unemployment'/de OR 'domestic violence'/de OR 'battered woman'/de OR 'family violence'/de OR 'partner violence'/exp OR 'family conflict'/exp OR 'social status'/de OR (((social* OR socioeconom* OR socio-econom* OR Socio-cultur*) NEAR/6 (determinant* OR status OR differen* OR correlat* OR predict* OR disadvantage* OR indicator* OR risk-factor* OR level* OR variable* OR vulnerab*)) OR (individual* NEAR/6 socioeconom* NEAR/6 (factor* OR determinant*)) OR divorce* OR low-school* OR (education* NEAR/3 (status* OR level OR matern* OR mother*)) OR unemploy* OR unmarried OR singlehood OR single-mother* OR ((domestic* OR famil* OR partner* OR spous* OR marital OR marriage OR household* OR house-hold*) NEAR/3 (status* OR violen* OR conflict* OR problem*))):ab,ti)
Perinatal outcomes	('fetus mortality'/exp OR 'fetus death'/de OR 'infant mortality'/de OR 'perinatal mortality'/exp OR 'low birth weight'/exp OR 'intrauterine growth retardation'/de OR 'immature and premature labor'/de OR (((fetus OR fetal OR foetus OR foetal OR perinatal* OR newborn* OR new-born* OR neonat* OR infant* OR antepart* OR antenatal* OR perinatal*) NEAR/3 (mortalit* OR surviv* OR fatal* OR death*)) OR (small NEAR/3 (date OR gestation*)) OR sga OR lbw OR vlbw OR elbw OR (low NEAR/3 (birth-weight* OR birthweight*)) OR ((intrauterin* OR intra-uterin* OR fetus OR fetal OR foetus OR foetal) NEAR/6 (retard* OR restrict*)) OR IUGR OR preterm* OR pre-term* OR ((prematur* OR immature OR dysmatur*) NEAR/3 (birth* OR childbirth OR born OR neonat* OR infan* OR labor OR labour))):ab,ti)
Population	('pregnancy'/exp OR 'pregnant woman'/exp OR 'prenatal period'/exp OR 'mother'/de OR 'expectant mother'/de OR 'maternal behavior'/de OR (pregnan* OR prenatal* OR antenatal* OR mother* OR maternal*):ab,ti)
Study design	('cohort analysis'/exp OR 'longitudinal study'/de OR 'retrospective study'/de OR 'prospective study'/de OR register/de OR 'factual database'/de OR (cohort* OR longitudinal* OR retrospectiv* OR prospectiv* OR register OR database*):ab,ti) NOT ([Conference Abstract]/lim OR [Letter]/lim OR [Note]/lim OR [Editorial]/lim) AND [english]/lim

Study selection

All articles identified with the database searches will be uploaded or manually entered into EndNote X8.2 reference management software (Thomson Reuters, New York City, NY). First, duplicates will be removed automatically and manually. Two reviewers (alternately LAD, MWG, or LM) will independently screen titles and abstracts of the retrieved articles to assess eligibility for inclusion. After the initial selection, full-texts of potentially eligible articles will be obtained and screened to assess eligibility for final inclusion. Disagreements will be resolved through consultation of a third reviewer (LCMB).

Data extraction

Two reviewers (alternately LAD, MWG, or LM) will independently extract relevant data from the included articles using a predefined data extraction form. This data extraction form will be piloted using the first five eligible articles and modified if required. The following data will be extracted from each article: author, publication year, study design, study setting, study period, number of included participants, study population characteristics, definition and measurements of the studied non-medical determinant(s), crude and adjusted effect estimates (i.e. odds ratio (OR) or relative risk), covariates used for adjustment, reported limitations, and key conclusions. If relevant information cannot be retrieved from the published articles, the authors of the manuscript will be contacted to request additional data.

For peer review only

Risk of bias assessment

To determine the risk of bias of the included studies, the Newcastle-Ottawa Scale (NOS) will be used.[23] The NOS is a validated tool for assessing the risk of bias among studies with an observational design. This tool is a commonly used tool for assessment of risk of bias and is considered the most practical tool in use compared to other tools.[24 25] Two reviewers (alternately LAD, MWG, or LM) will independently assess the risk of bias of the included articles. Disagreements will be resolved through discussion with a third reviewer (LCMB).

Data analysis

ORs and 95% confidence intervals will be extracted from the included articles to express the effect of the associations between the studied non-medical determinants and perinatal mortality, preterm birth, and SGA. Data presented in other effect measures (e.g. relative risks, beta-coefficients) will be converted into ORs if possible. Publication bias will be evaluated using funnel plots and corresponding Begg's and Egger's tests. The degree of heterogeneity among the included articles will be examined using the I^2 statistic. The pooled effects will be calculated separately for each association found between one of the outcome measures and the studied non-medical determinants, using a random effects model. All analyses will be performed using R package 'metafor'.

In addition to the meta-analysis, the population-attributable fraction (PAF) will be calculated based on the effect estimates of the meta-analysis. The PAF will be used to estimate the contribution of each non-medical determinant to the burden of every single outcome measure (i.e. perinatal mortality, preterm birth, and SGA) within the European context. The following formula will be used to calculate the PAF:

$$PAF = \frac{P_{pop} \times (OR - 1)}{P_{pop} \times (OR - 1) + 1}$$

In which, P_{pop} is the proportion of the population (i.e. pregnant women) exposed (i.e. to the specific non-medical determinant) and OR is the pooled odds ratio of the outcome in the exposed versus the non-exposed group.

Ethics and dissemination: No formal ethical assessment or informed consent is required for the purpose of this study. In accordance with the PRISMA-P guidelines, the study is registered with PROSPERO (CRD42018056105, November 12, 2018).[22 26] The findings of this study will be summarised in a manuscript, which will be submitted for publication in a peer-reviewed scientific journal.

Authors' contribution: EAPS, LCMB and MWG conceived this systematic review with meta-analysis. LAD and MWG developed and drafted the protocol. MWG registered the protocol in PROSPERO. EAPS, LCMB and LM reviewed the protocol and provided extensive feedback. All authors read and approved the final manuscript.

Funding statement: This research received no specific grant from any funding agency in the public, commercial or not-for-profit sectors.

Competing interests statement: None declared.

Acknowledgments: None.

For peer review only

References

1. Blencowe H, Cousens S, Oestergaard MZ, et al. National, regional, and worldwide estimates of preterm birth rates in the year 2010 with time trends since 1990 for selected countries: a systematic analysis and implications. *Lancet* 2012;379(9832):2162-72.
2. Lee ACC, Katz J, Blencowe H, et al. National and regional estimates of term and preterm babies born small for gestational age in 138 low-income and middle-income countries in 2010. *Lancet Glob Health* 2013;1(1):e26-e36.
3. Liu L, Johnson HL, Cousens S, et al. Global, regional, and national causes of child mortality: an updated systematic analysis for 2010 with time trends since 2000. *Lancet* 2012;379(9832):2151-61.
4. Vos AA, Posthumus AG, Bonsel GJ, Steegers EAP, Denktaş S. Deprived neighborhoods and adverse perinatal outcome: a systematic review and meta-analysis. *Acta Obstet Gynecol Scand* 2014;93(8):727-40.
5. Gray R, Bonellie SR, Chalmers J, Greer I, Jarvis S, Williams C. Social inequalities in preterm birth in Scotland 1980–2003: findings from an area-based measure of deprivation. *BJOG* 2008;115(1):82-90.
6. Matijasevich A, Victora CG, Lawlor DA, et al. Association of socioeconomic position with maternal pregnancy and infant health outcomes in birth cohort studies from Brazil and the UK. *J Epidemiol Community Health* 2012;66(2):127-35.
7. Weightman AL, Morgan HE, Shepherd MA, Kitcher H, Roberts C, Dunstan FD. Social inequality and infant health in the UK: systematic review and meta-analyses. *BMJ Open* 2012;2(3):e000964.
8. Goedhart G, Van Eijsden M, Van Der Wal MF, Bonsel GJ. Ethnic differences in preterm birth and its subtypes: the effect of a cumulative risk profile. *BJOG* 2008;115(6):710-9.
9. Timmermans S, Bonsel GJ, Steegers-Theunissen RPM, et al. Individual accumulation of heterogeneous risks explains perinatal inequalities within deprived neighbourhoods. *Eur J Epidemiol* 2011;26(2):165-80.
10. Agyemang C, Vrijkotte TGM, Droomers M, Van der Wal MF, Bonsel GJ, Stronks K. The effect of neighbourhood income and deprivation on pregnancy outcomes in Amsterdam, The Netherlands. *J Epidemiol Community Health* 2009;63(9):755-60.
11. Flenady V, Koopmans L, Middleton P, et al. Major risk factors for stillbirth in high-income countries: a systematic review and meta-analysis. *Lancet* 2011;377(9774):1331-40.
12. Barker DJP. The origins of the developmental origins theory. *J Intern Med* 2007;261(5):412-7.
13. Jansen PW, Tiemeier H, Jaddoe VWV, et al. Explaining educational inequalities in preterm birth: the generation r study. *Arch Dis Child Fetal Neonatal Ed* 2009;94(1):F28-F34.
14. Oftedal A-M, Busterud K, Irgens LM, Haug K, Rasmussen S. Socio-economic risk factors for preterm birth in Norway 1999–2009. *Scand J Public Health* 2016;44(6):587-92.
15. Saurel-Cubizolles MJ, Zeitlin J, Lelong N, Papiernik E, Di Renzo GC, Bréart G. Employment, working conditions, and preterm birth: results from the Europop case-control survey. *J Epidemiol Community Health* 2004;58(5):395-401.
16. Smeeton NC, Rona RJ, Dobson P, Cochrane R, Wolfe C. Assessing the determinants of stillbirths and early neonatal deaths using routinely collected data in an inner city area. *BMC Med* 2004;2(1):27.
17. Sundquist J, Sundquist K, Johansson S-E, Li X, Winkleby M. Mothers, places and small for gestational age births: a cohort study. *Arch Dis Child* 2011;96(4):380-5.
18. Jansen PW, Tiemeier H, Verhulst FC, et al. Employment status and the risk of pregnancy complications: the Generation R Study. *Occup Environ Med* 2010;67(6):387-94.

For peer review only

19. Morales-Suárez-Varela M, Kaerlev L, Zhu JL, et al. Unemployment and pregnancy outcomes: a study within the Danish National Birth Cohort. *Scand J Public Health* 2011;39(5):449-56.
20. Stringhini S, Carmeli C, Jokela M, et al. Socioeconomic status and the 25× 25 risk factors as determinants of premature mortality: a multicohort study and meta-analysis of 1· 7 million men and women. *Lancet* 2017;389(10075):1229-37.
21. Naghavi M, Wang H, Lozano R, et al. Global, regional, and national age-sex specific all-cause and cause-specific mortality for 240 causes of death, 1990-2013: a systematic analysis for the Global Burden of Disease Study 2013. *Lancet* 2015;385(9963):117-71.
22. Shamseer L, Moher D, Clarke M, et al. Preferred reporting items for systematic review and meta-analysis protocols (PRISMA-P) 2015: elaboration and explanation. *BMJ* 2015;350:g7647.
23. Wells GA, Shea B, O'Connell D, et al. The Newcastle-Ottawa Scale (NOS) for assessing the quality of nonrandomised studies in meta-analyses. http://www.ohri.ca/programs/clinical_epidemiology/oxford.asp.
24. Deeks JJ, Dinnes J, D'Amico R, et al. Evaluating non-randomised intervention studies. *Health Technol Assess* 2003;7(27):iii-x, 1-173.
25. Higgins JPT, Green S. Cochrane handbook for systematic reviews of interventions 5.1.0. March 2011. http://handbook-5-1.cochrane.org/.
26. Moher D, Shamseer L, Clarke M, et al. Preferred reporting items for systematic review and meta-analysis protocols (PRISMA-P) 2015 statement. *Syst Rev* 2015;4(1):1.

For peer review only

Supplementary file 1. PRISMA-P Guidelines

PRISMA-P (Preferred Reporting Items for Systematic review and Meta-Analysis Protocols) 2015 checklist: recommended items to address in a systematic review protocol*

Section and topic	Item No	Checklist item
**ADMINISTRATIVE INFORMATION**		
Title:		
Identification	1a	Identify the report as a protocol of a systematic review
Update	1b	If the protocol is for an update of a previous systematic review, identify as such
Registration	2	If registered, provide the name of the registry (such as PROSPERO) and registration number
Authors:		
Contact	3a	Provide name, institutional affiliation, e-mail address of all protocol authors; provide physical mailing address of corresponding author
Contributions	3b	Describe contributions of protocol authors and identify the guarantor of the review
Amendments	4	If the protocol represents an amendment of a previously completed or published protocol, identify as such and list changes; otherwise, state plan for documenting important protocol amendments
Support:		
Sources	5a	Indicate sources of financial or other support for the review
Sponsor	5b	Provide name for the review funder and/or sponsor
Role of sponsor or funder	5c	Describe roles of funder(s), sponsor(s), and/or institution(s), if any, in developing the protocol
**INTRODUCTION**		
Rationale	6	Describe the rationale for the review in the context of what is already known
Objectives	7	Provide an explicit statement of the question(s) the review will address with reference to participants, interventions, comparators, and outcomes (PICO)
**METHODS**		
Eligibility criteria	8	Specify the study characteristics (such as PICO, study design, setting, time frame) and report characteristics (such as years considered, language, publication status) to be used as criteria for eligibility for the review
Information sources	9	Describe all intended information sources (such as electronic databases, contact with study authors, trial registers or other grey literature sources) with planned dates of coverage

For peer review only

Search strategy	10	Present draft of search strategy to be used for at least one electronic database, including planned limits, such that it could be repeated
Study records:		
Data management	11a	Describe the mechanism(s) that will be used to manage records and data throughout the review
Selection process	11b	State the process that will be used for selecting studies (such as two independent reviewers) through each phase of the review (that is, screening, eligibility and inclusion in meta-analysis)
Data collection process	11c	Describe planned method of extracting data from reports (such as piloting forms, done independently, in duplicate), any processes for obtaining and confirming data from investigators
Data items	12	List and define all variables for which data will be sought (such as PICO items, funding sources), any pre-planned data assumptions and simplifications
Outcomes and prioritization	13	List and define all outcomes for which data will be sought, including prioritization of main and additional outcomes, with rationale
Risk of bias in individual studies	14	Describe anticipated methods for assessing risk of bias of individual studies, including whether this will be done at the outcome or study level, or both; state how this information will be used in data synthesis
Data synthesis	15a	Describe criteria under which study data will be quantitatively synthesised
	15b	If data are appropriate for quantitative synthesis, describe planned summary measures, methods of handling data and methods of combining data from studies, including any planned exploration of consistency (such as I^2, Kendall's τ)
	15c	Describe any proposed additional analyses (such as sensitivity or subgroup analyses, meta-regression)
	15d	If quantitative synthesis is not appropriate, describe the type of summary planned
Meta-bias(es)	16	Specify any planned assessment of meta-bias(es) (such as publication bias across studies, selective reporting within studies)
Confidence in cumulative evidence	17	Describe how the strength of the body of evidence will be assessed (such as GRADE)

*** It is strongly recommended that this checklist be read in conjunction with the PRISMA-P Explanation and Elaboration (cite when available) for important clarification on the items. Amendments to a review protocol should be tracked and dated. The copyright for PRISMA-P (including checklist) is held by the PRISMA-P Group and is distributed under a Creative Commons Attribution Licence 4.0.**

From: Shamseer L, Moher D, Clarke M, Ghersi D, Liberati A, Petticrew M, Shekelle P, Stewart L, PRISMA-P Group. Preferred reporting items for systematic review and meta-analysis protocols (PRISMA-P) 2015: elaboration and explanation. BMJ. 2015 Jan 2;349(jan02 1):g7647.

For peer review only

Research Checklist

PRISMA-P (Preferred Reporting Items for Systematic review and Meta-Analysis Protocols) 2015 checklist: recommended items to address in a systematic review protocol*

Section and topic	Item No	Checklist item	(Page No.#)
**ADMINISTRATIVE INFORMATION**			
Title:			
Identification	1a	Identify the report as a protocol of a systematic review	1
Update	1b	If the protocol is for an update of a previous systematic review, identify as such	Not applicable
Registration	2	If registered, provide the name of the registry (such as PROSPERO) and registration number	2
Authors:			
Contact	3a	Provide name, institutional affiliation, e-mail address of all protocol authors; provide physical mailing address of corresponding author	1
Contributions	3b	Describe contributions of protocol authors and identify the guarantor of the review	8
Amendments	4	If the protocol represents an amendment of a previously completed or published protocol, identify as such and list changes; otherwise, state plan for documenting important protocol amendments	Not applicable
Support:			
Sources	5a	Indicate sources of financial or other support for the review	8
Sponsor	5b	Provide name for the review funder and/or sponsor	
Role of sponsor or funder	5c	Describe roles of funder(s), sponsor(s), and/or institution(s), if any, in developing the protocol	
**INTRODUCTION**			
Rationale	6	Describe the rationale for the review in the context of what is already known	3
Objectives	7	Provide an explicit statement of the question(s) the review will address with reference to participants, interventions, comparators, and outcomes (PICO)	3
**METHODS**			
Eligibility criteria	8	Specify the study characteristics (such as PICO, study design, setting, time frame) and report characteristics (such as years considered, language, publication status) to be used as criteria for eligibility for the review	4

For peer review only

Information sources	9	Describe all intended information sources (such as electronic databases, contact with study authors, trial registers or other grey literature sources) with planned dates of coverage	4-5
Search strategy	10	Present draft of search strategy to be used for at least one electronic database, including planned limits, such that it could be repeated	5 (table 1)
Study records:			
Data management	11a	Describe the mechanism(s) that will be used to manage records and data throughout the review	5
Selection process	11b	State the process that will be used for selecting studies (such as two independent reviewers) through each phase of the review (that is, screening, eligibility and inclusion in meta-analysis)	5
Data collection process	11c	Describe planned method of extracting data from reports (such as piloting forms, done independently, in duplicate), any processes for obtaining and confirming data from investigators	5
Data items	12	List and define all variables for which data will be sought (such as PICO items, funding sources), any pre-planned data assumptions and simplifications	5
Outcomes and prioritization	13	List and define all outcomes for which data will be sought, including prioritization of main and additional outcomes, with rationale	4
Risk of bias in individual studies	14	Describe anticipated methods for assessing risk of bias of individual studies, including whether this will be done at the outcome or study level, or both; state how this information will be used in data synthesis	6
Data synthesis	15a	Describe criteria under which study data will be quantitatively synthesised	6
	15b	If data are appropriate for quantitative synthesis, describe planned summary measures, methods of handling data and methods of combining data from studies, including any planned exploration of consistency (such as I^2, Kendall's τ)	6
	15c	Describe any proposed additional analyses (such as sensitivity or subgroup analyses, meta-regression)	6
	15d	If quantitative synthesis is not appropriate, describe the type of summary planned	6
Meta-bias(es)	16	Specify any planned assessment of meta-bias(es) (such as publication bias across studies, selective reporting within studies)	6
Confidence in cumulative evidence	17	Describe how the strength of the body of evidence will be assessed (such as GRADE)	6

*** It is strongly recommended that this checklist be read in conjunction with the PRISMA-P Explanation and Elaboration (cite when available) for important clarification on the items. Amendments to a review protocol should be tracked and dated.** **The copyright for PRISMA-P (including checklist) is held by the PRISMA-P Group and is distributed under a Creative Commons Attribution Licence 4.0.**

From: Shamseer L, Moher D, Clarke M, Ghersi D, Liberati A, Petticrew M, Shekelle P, Stewart L, PRISMA-P Group. Preferred reporting items for systematic review and meta-analysis protocols (PRISMA-P) 2015: elaboration and explanation. BMJ. 2015 Jan 2;349(jan02 1):g7647.

For peer review only

BMJ Open

Non-medical determinants of perinatal health: protocol for a systematic review with meta-analysis

Journal:	*BMJ Open*
Manuscript ID	bmjopen-2019-031437.R1
Article Type:	Protocol
Date Submitted by the Author:	01-Aug-2019
Complete List of Authors:	Daalderop, Leonie; Erasmus MC, Obstetrics and Gynaecology<br>de Groot, Marjolein; Erasmus MC, Obstetrics and Gyneaecology<br>van der Meer, Lindsey; Erasmus MC, Obstetrics and Gynaecology<br>Steegers, Eric; Erasmus MC,<br>Bertens, Loes; Erasmus MC, Department of Obstetrics and Gynaecology
**Primary Subject Heading**:	Obstetrics and gynaecology
Secondary Subject Heading:	Public health
Keywords:	Perinatal health, social determinants of health, systematic review, meta-analysis, non-medical determinants

SCHOLARONE™
Manuscripts

For peer review only - http://bmjopen.bmj.com/site/about/guidelines.xhtml

Non-medical determinants of perinatal health: protocol for a systematic review with meta-analysis

Leonie A Daalderop[1 2], Marjolein W de Groot[1], Lindsey van der Meer[1], Eric A P Steegers[1], Loes C M Bertens[1]

[1]Department of Obstetrics and Gynaecology, Erasmus MC, University Medical Centre Rotterdam, PO Box 2040, 3000 CA, Rotterdam, The Netherlands

[2]Dutch Research Institute for Transitions, Erasmus University Rotterdam, Postbus 1738, 3000 DR Rotterdam, The Netherlands

Corresponding author: Leonie A. Daalderop

Address: Erasmus University Medical Centre, Department of Obstetrics and Gynaecology, PO Box 2040, 3000 CA, Rotterdam, The Netherlands

Telephone number: +31 (0)10 703 15 45

E-mail address: l.daalderop@erasmusmc.nl

Word count: 1,354 words.

Key words: Perinatal health, non-medical determinants, social determinants of health, systematic review, meta-analysis

For peer review only

ABSTRACT

Introduction: Research focusing on the associations between non-medical determinants and unfavourable perinatal health outcomes is increasing. Despite increasing knowledge on this theme, it still remains unclear to what extent social, environmental, and lifestyle factors contribute to these unfavourable outcomes. Therefore, we aim to provide a systematic review, preferably with meta-analysis, in order to provide insight into the associations between non-medical determinants and perinatal mortality, preterm birth, and small for gestational age (SGA).

Methods and analysis: Observational studies performed in European countries, studying the associations between non-medical determinants and unfavourable perinatal health outcomes will be included. Primary outcomes of interest are: perinatal mortality, preterm birth, and SGA. To retrieve potential eligible articles, a systematic literature search was performed in the online databases MEDLINE, EMBASE, Web of Science, Cochrane, and Google Scholar on October 5, 2018. Additionally, a reference list check and citation search will be performed. Data of the included articles will be extracted using a standardised and piloted data-extraction form. Risk of bias will be assessed using the Newcastle-Ottawa Scale. The study selection and data extraction process will be performed by two reviewers independently. Disagreements will be resolved through discussion with a third reviewer. The pooled effects will be calculated separately for each association found between one of the outcome measures and the non-medical determinants, using a random effects model. Heterogeneity of the studies will be assessed using the I^2 statistic.

Ethics and dissemination: No ethical approval is necessary for a systematic review with meta-analysis. The findings will be published in a peer-reviewed journal.

Prospero registration number: CRD42018056105

ARTICLE SUMMARY

Strengths and limitations of this study

- This systematic review with meta-analysis will provide a comprehensive overview of the existing literature on the associations between non-medical determinants and perinatal mortality, preterm birth, and SGA.
- The results of the meta-analysis can highlight the relevance of the studied non-medical determinants as well as the need to develop preventive strategies aimed at diminishing unfavourable perinatal health outcomes.
- This systematic review focuses on studies performed in European countries with more or less comparable health care systems, which limits the generalisability of our findings to countries outside Europe with different health care systems and/or a different welfare state.

For peer review only - http://bmjopen.bmj.com/site/about/guidelines.xhtml

INTRODUCTION

Preterm birth and being small for gestational age (SGA) affect 11 to 27 percent of all live births worldwide and are the leading causes of perinatal mortality.[1-3] Beside the negative impact of well-known medical and obstetric risk factors, risk accumulation of non-medical determinants related to a person's social status and environmental surroundings can explain additional variation in the occurrence of unfavourable perinatal health outcomes (i.e. perinatal mortality, preterm birth, and SGA).[4-9] These factors encompasses for instance educational level, marital status, household income and housing conditions. The negative impact of non-medical determinants on perinatal health outcomes extends into adulthood, with long-term health consequences for the affected children and therewith major implications for public health.[10]

During the past decade, many studies have tried to identify the effects of non-medical determinants on unfavourable perinatal health outcomes.[4 6-9 11-19] Several studies reported non-medical determinants as risk factors for unfavourable perinatal health outcomes.[4 6 7 11-14] Additionally, studies indicate that the negative impact of non-medical determinants is often the result of clustering of two or more risk factors.[8 9] However, many other studies do not show associations between non-medical determinants and unfavourable perinatal health outcomes.[15-19] Furthermore, research on this topic often focuses on the associations between a limited number of non-medical determinants and a single outcome measure, such as preterm delivery, low birth weight, or SGA. Non-medical determinants which are often related to a person's social status are highly associated. Therefore, it is important to investigate these different non-medical determinants together, as well as the way they influence each other. This together with the global burden of both non-medical determinants and unfavourable perinatal health outcomes, a comprehensive overview of relevant non-medical determinants and the way they affect perinatal health outcomes is necessary to deliver the best possible care for vulnerable pregnant women.[3 20 21]

Therefore, we aim to provide a systematic review, preferably with meta-analysis, in order to investigate the impact of a broad range of non-medical determinants on unfavourable perinatal health outcomes. This can help scientists as well as healthcare providers to thoroughly understand the role of non-medical determinants with regard to unfavourable perinatal health outcomes.

For peer review only

For peer review only - http://bmjopen.bmj.com/site/about/guidelines.xhtml

METHODS AND ANALYSIS

The PRISMA-P (Preferred Reporting Items for Systematic Reviews and Meta-analyses Protocols) guidelines were used to guide the reporting of this systematic review protocol (supplementary file 1).[22] The initial search was performed on October 5, 2018 and the study was registered with the PROSPERO prospective register of systematic reviews on November 12, 2018 (CRD42018056105). The expected finalisation of the systematic review with meta-analysis is end 2019.

Eligibility criteria

Studies will be selected according to the criteria outlined below.

Study design and participants

We will include all observational studies (i.e. cohort, case-control, and cross-sectional studies) reporting on pregnant women that study the associations between exposure to non-medical determinants during pregnancy and unfavourable perinatal health outcomes (i.e. fetal complications during pregnancy, labour, and/or delivery). Exposures have to be measured at the individual level. Studies performed in European countries are eligible for inclusion, as these are countries with a more homogeneous population and, more or less, comparable health care systems. Experimental studies, case reports, editorials, commentary, and clinical guidelines will be excluded. Additionally, systematic reviews and meta-analyses will be excluded; however their reference lists will be screened for potential eligible articles. Only articles written in English will be included.

Determinants of interest

This systematic review will focus on non-medical determinants that may influence perinatal health outcomes. Non-medical determinants that will be taken into account were pre-specified. These non-medical determinants are:

- Socioeconomic status (SES);
- Marital status;
- Domestic violence;
- Employment status;
- Educational level.

After title and abstract screening, another three non-medical determinants were identified which were not specified on beforehand. These non-medical determinants were often investigated in relation to unfavourable perinatal health outcomes and therefore we decided to add these determinants to our systematic review.

- Housing (e.g. place of residence, housing conditions);
- Income;
- Paternal determinants (e.g. non-medical determinants related to the father).

Outcomes

The primary outcomes are:

- Perinatal mortality: death occurring between 22 weeks of gestational age and 7 days after birth;
- Preterm birth: delivery of a live-born baby before 37 completed weeks of gestation;
- SGA: birth weight below the 10th centile adjusted for ethnicity, parity, gestational age, and gender.

Secondary outcomes are:

- Neonatal mortality: death of a baby occurring within the first 28 days of life;
- Low birth weight: birth weight below 2,500 grams.

For peer review only - http://bmjopen.bmj.com/site/about/guidelines.xhtml

It is possible that studies have used outcome definitions that differ from the ones defined above. Studies are in that case still eligible to be included into the systematic review. For the meta-analyses of the effects per outcome, only the effects from studies with a sufficient amount of overlap in the used definitions will be pooled.

Information sources and search strategy

The electronic online databases MEDLINE, EMBASE, Web of Science, Cochrane, and Google Scholar were initially searched on October 5, 2018. The electronic search yielded 4,980 references. The search strategy will be supplemented by screening reference lists and performing a citation search of the included articles. Also, the search will be updated after finalisation of the data extraction process. A search strategy was developed for EMBASE (table 1), using Emtree terms and free text terms related to non-medical determinants, perinatal health outcomes, pregnant women, and the study design. The search strategy was amended for use in other databases. No limitations will be applied regarding publication date.

Table 1. Search strategy in EMBASE

Element	Search items
Non-medical determinants	('social determinants of health'/exp OR 'marriage'/de OR divorce/de OR 'female by marital status'/exp OR 'educational status'/exp OR 'unemployment'/de OR 'domestic violence'/de OR 'battered woman'/de OR 'family violence'/de OR 'partner violence'/exp OR 'family conflict'/exp OR 'social status'/de OR (((social* OR socioeconom* OR socio-econom* OR Socio-cultur*) NEAR/6 (determinant* OR status OR differen* OR correlat* OR predict* OR disadvantage* OR indicator* OR risk-factor* OR level* OR variable* OR vulnerab*)) OR (individual* NEAR/6 socioeconom* NEAR/6 (factor* OR determinant*)) OR divorce* OR low-school* OR (education* NEAR/3 (status* OR level OR matern* OR mother*)) OR unemploy* OR unmarried OR singlehood OR single-mother* OR ((domestic* OR famil* OR partner* OR spous* OR marital OR marriage OR household* OR house-hold*) NEAR/3 (status* OR violen* OR conflict* OR problem*))):ab,ti)
Perinatal outcomes	('fetus mortality'/exp OR 'fetus death'/de OR 'infant mortality'/de OR 'perinatal mortality'/exp OR 'low birth weight'/exp OR 'intrauterine growth retardation'/de OR 'immature and premature labor'/de OR (((fetus OR fetal OR foetus OR foetal OR perinatal* OR newborn* OR new-born* OR neonat* OR infant* OR antepart* OR antenatal* OR perinatal*) NEAR/3 (mortalit* OR surviv* OR fatal* OR death*)) OR (small NEAR/3 (date OR gestation*)) OR sga OR lbw OR vlbw OR elbw OR (low NEAR/3 (birth-weight* OR birthweight*)) OR ((intrauterin* OR intra-uterin* OR fetus OR fetal OR foetus OR foetal) NEAR/6 (retard* OR restrict*)) OR IUGR OR preterm* OR pre-term* OR ((prematur* OR immature OR dysmatur*) NEAR/3 (birth* OR childbirth OR born OR neonat* OR infan* OR labor OR labour))):ab,ti)
Population	('pregnancy'/exp OR 'pregnant woman'/exp OR 'prenatal period'/exp OR 'mother'/de OR 'expectant mother'/de OR 'maternal behavior'/de OR (pregnan* OR prenatal* OR antenatal* OR mother* OR maternal*):ab,ti)
Study design	('cohort analysis'/exp OR 'longitudinal study'/de OR 'retrospective study'/de OR 'prospective study'/de OR register/de OR 'factual database'/de OR (cohort* OR longitudinal* OR retrospectiv* OR prospectiv* OR register OR database*):ab,ti) NOT ([Conference Abstract]/lim OR [Letter]/lim OR [Note]/lim OR [Editorial]/lim) AND [english]/lim

Study selection

All articles identified with the database searches will be uploaded or manually entered into EndNote X8.2 reference management software (Thomson Reuters, New York City, NY). First, duplicates will be removed automatically and manually. Two reviewers (alternately LAD, MWG, or LM) will independently screen titles and abstracts of the retrieved articles to assess eligibility for inclusion. After the initial selection, full-texts of potentially eligible articles will be obtained and screened to assess eligibility for final inclusion. Disagreements will be resolved through consultation of a third reviewer (LCMB).

For peer review only - http://bmjopen.bmj.com/site/about/guidelines.xhtml

Data extraction

Two reviewers (alternately LAD, MWG, or LM) will independently extract relevant data from the included articles using a predefined data extraction form. This data extraction form will be piloted using the first five eligible articles and modified if required. The following data will be extracted from each article: author, publication year, study design, study setting, study period, number of included participants, study population characteristics, definition and measurements of the studied non-medical determinant(s), crude and adjusted effect estimates (i.e. odds ratio (OR) or relative risk (RR)), covariates used for adjustment, reported limitations, and key conclusions. If relevant information cannot be retrieved from the published articles, the authors of the manuscript will be contacted to request additional data.

Risk of bias assessment

To determine the risk of bias of the included studies, the Newcastle-Ottawa Scale (NOS) will be used.[23] The NOS is a validated tool for assessing the risk of bias among studies with an observational design. This tool is a commonly used tool for assessment of risk of bias and is considered the most practical tool in use compared to other tools.[24 25] Two reviewers (alternately LAD, MWG, or LM) will independently assess the risk of bias of the included articles. Disagreements will be resolved through discussion with a third reviewer (LCMB).

Data analysis

ORs and 95% confidence intervals will be extracted from the included articles to express the effect of the associations between the studied non-medical determinants and perinatal mortality, preterm birth, and SGA. Data presented in other effect measures (e.g. RRs, beta-coefficients) will be converted into ORs when possible. Publication bias will be evaluated using funnel plots and corresponding Begg's and Egger's tests. The degree of heterogeneity among the included articles will be examined using the I^2 statistic. The pooled effects will be calculated when there is sufficient overlap among the used definitions among the included studies. When a meta-analysis can be performed, the pooled effect estimates will be calculated separately for each association found between one of the outcome measures and the studied non-medical determinants, using a random effects model. The meta-analysis will be performed with both the unadjusted and adjusted estimates, due to the expected heterogeneity in the degree of adjustment. All analyses will be performed using R package 'metafor'.

Patient and Public Involvement: No patients and/or public will be involved in this study.

Ethics and dissemination: No formal ethical assessment or informed consent is required for the purpose of this study. In accordance with the PRISMA-P guidelines, the study is registered with PROSPERO (CRD42018056105, November 12, 2018).[22 26] The findings of this study will be summarised in a manuscript, which will be submitted for publication in a peer-reviewed scientific journal.

Authors' contribution: EAPS, LCMB and MWG conceived this systematic review with meta-analysis. LAD and MWG developed and drafted the protocol. MWG registered the protocol in PROSPERO. EAPS, LCMB and LM reviewed the protocol and provided extensive feedback. All authors read and approved the final manuscript.

Funding statement: This research received no specific grant from any funding agency in the public, commercial or not-for-profit sectors.

Competing interests statement: None declared.

For peer review only - http://bmjopen.bmj.com/site/about/guidelines.xhtml

Acknowledgments: None.

References

1. Blencowe H, Cousens S, Oestergaard MZ, et al. National, regional, and worldwide estimates of preterm birth rates in the year 2010 with time trends since 1990 for selected countries: a systematic analysis and implications. *Lancet* 2012;379(9832):2162-72.
2. Lee ACC, Katz J, Blencowe H, et al. National and regional estimates of term and preterm babies born small for gestational age in 138 low-income and middle-income countries in 2010. *Lancet Glob Health* 2013;1(1):e26-e36.
3. Liu L, Johnson HL, Cousens S, et al. Global, regional, and national causes of child mortality: an updated systematic analysis for 2010 with time trends since 2000. *Lancet* 2012;379(9832):2151-61.
4. Vos AA, Posthumus AG, Bonsel GJ, Steegers EAP, Denktaş S. Deprived neighborhoods and adverse perinatal outcome: a systematic review and meta-analysis. *Acta Obstet Gynecol Scand* 2014;93(8):727-40.
5. Gray R, Bonellie SR, Chalmers J, Greer I, Jarvis S, Williams C. Social inequalities in preterm birth in Scotland 1980–2003: findings from an area-based measure of deprivation. *BJOG* 2008;115(1):82-90.
6. Matijasevich A, Victora CG, Lawlor DA, et al. Association of socioeconomic position with maternal pregnancy and infant health outcomes in birth cohort studies from Brazil and the UK. *J Epidemiol Community Health* 2012;66(2):127-35.
7. Weightman AL, Morgan HE, Shepherd MA, Kitcher H, Roberts C, Dunstan FD. Social inequality and infant health in the UK: systematic review and meta-analyses. *BMJ Open* 2012;2(3):e000964.
8. Goedhart G, Van Eijsden M, Van Der Wal MF, Bonsel GJ. Ethnic differences in preterm birth and its subtypes: the effect of a cumulative risk profile. *BJOG* 2008;115(6):710-9.
9. Timmermans S, Bonsel GJ, Steegers-Theunissen RPM, et al. Individual accumulation of heterogeneous risks explains perinatal inequalities within deprived neighbourhoods. *Eur J Epidemiol* 2011;26(2):165-80.
10. Barker DJP. The origins of the developmental origins theory. *J Intern Med* 2007;261(5):412-7.
11. Agyemang C, Vrijkotte TGM, Droomers M, Van der Wal MF, Bonsel GJ, Stronks K. The effect of neighbourhood income and deprivation on pregnancy outcomes in Amsterdam, The Netherlands. *J Epidemiol Community Health* 2009;63(9):755-60.
12. Flenady V, Koopmans L, Middleton P, et al. Major risk factors for stillbirth in high-income countries: a systematic review and meta-analysis. *Lancet* 2011;377(9774):1331-40.
13. Jansen PW, Tiemeier H, Jaddoe VWV, et al. Explaining educational inequalities in preterm birth: the generation r study. *Arch Dis Child Fetal Neonatal Ed* 2009;94(1):F28-F34.
14. Oftedal A-M, Busterud K, Irgens LM, Haug K, Rasmussen S. Socio-economic risk factors for preterm birth in Norway 1999–2009. *Scand J Public Health* 2016;44(6):587-92.
15. Saurel-Cubizolles MJ, Zeitlin J, Lelong N, Papiernik E, Di Renzo GC, Bréart G. Employment, working conditions, and preterm birth: results from the Europop case-control survey. *J Epidemiol Community Health* 2004;58(5):395-401.
16. Smeeton NC, Rona RJ, Dobson P, Cochrane R, Wolfe C. Assessing the determinants of stillbirths and early neonatal deaths using routinely collected data in an inner city area. *BMC Med* 2004;2(1):27.
17. Sundquist J, Sundquist K, Johansson S-E, Li X, Winkleby M. Mothers, places and small for gestational age births: a cohort study. *Arch Dis Child* 2011;96(4):380-5.

For peer review only

For peer review only - http://bmjopen.bmj.com/site/about/guidelines.xhtml

18. Jansen PW, Tiemeier H, Verhulst FC, et al. Employment status and the risk of pregnancy complications: the Generation R Study. *Occup Environ Med* 2010;67(6):387-94.
19. Morales-Suárez-Varela M, Kaerlev L, Zhu JL, et al. Unemployment and pregnancy outcomes: a study within the Danish National Birth Cohort. *Scand J Public Health* 2011;39(5):449-56.
20. Stringhini S, Carmeli C, Jokela M, et al. Socioeconomic status and the 25× 25 risk factors as determinants of premature mortality: a multicohort study and meta-analysis of 1· 7 million men and women. *Lancet* 2017;389(10075):1229-37.
21. Naghavi M, Wang H, Lozano R, et al. Global, regional, and national age-sex specific all-cause and cause-specific mortality for 240 causes of death, 1990-2013: a systematic analysis for the Global Burden of Disease Study 2013. *Lancet* 2015;385(9963):117-71.
22. Shamseer L, Moher D, Clarke M, et al. Preferred reporting items for systematic review and meta-analysis protocols (PRISMA-P) 2015: elaboration and explanation. *BMJ* 2015;350:g7647.
23. Wells GA, Shea B, O'Connell D, et al. The Newcastle-Ottawa Scale (NOS) for assessing the quality of nonrandomised studies in meta-analyses. http://www.ohri.ca/programs/clinical_epidemiology/oxford.asp.
24. Deeks JJ, Dinnes J, D'Amico R, et al. Evaluating non-randomised intervention studies. *Health Technol Assess* 2003;7(27):iii-x, 1-173.
25. Higgins JPT, Green S. Cochrane handbook for systematic reviews of interventions 5.1.0. March 2011. http://handbook-5-1.cochrane.org/.
26. Moher D, Shamseer L, Clarke M, et al. Preferred reporting items for systematic review and meta-analysis protocols (PRISMA-P) 2015 statement. *Syst Rev* 2015;4(1):1.

For peer review only

For peer review only - http://bmjopen.bmj.com/site/about/guidelines.xhtml

Supplementary file 1. PRISMA-P Guidelines

PRISMA-P (Preferred Reporting Items for Systematic review and Meta-Analysis Protocols) 2015 checklist: recommended items to address in a systematic review protocol*

Section and topic	Item No	Checklist item
**ADMINISTRATIVE INFORMATION**		
Title:		
Identification	1a	Identify the report as a protocol of a systematic review
Update	1b	If the protocol is for an update of a previous systematic review, identify as such
Registration	2	If registered, provide the name of the registry (such as PROSPERO) and registration number
Authors:		
Contact	3a	Provide name, institutional affiliation, e-mail address of all protocol authors; provide physical mailing address of corresponding author
Contributions	3b	Describe contributions of protocol authors and identify the guarantor of the review
Amendments	4	If the protocol represents an amendment of a previously completed or published protocol, identify as such and list changes; otherwise, state plan for documenting important protocol amendments
Support:		
Sources	5a	Indicate sources of financial or other support for the review
Sponsor	5b	Provide name for the review funder and/or sponsor
Role of sponsor or funder	5c	Describe roles of funder(s), sponsor(s), and/or institution(s), if any, in developing the protocol
**INTRODUCTION**		
Rationale	6	Describe the rationale for the review in the context of what is already known
Objectives	7	Provide an explicit statement of the question(s) the review will address with reference to participants, interventions, comparators, and outcomes (PICO)
**METHODS**		
Eligibility criteria	8	Specify the study characteristics (such as PICO, study design, setting, time frame) and report characteristics (such as years considered, language, publication status) to be used as criteria for eligibility for the review
Information sources	9	Describe all intended information sources (such as electronic databases, contact with study authors, trial registers or other grey literature sources) with planned dates of coverage

For peer review only

Search strategy	10	Present draft of search strategy to be used for at least one electronic database, including planned limits, such that it could be repeated
Study records:		
Data management	11a	Describe the mechanism(s) that will be used to manage records and data throughout the review
Selection process	11b	State the process that will be used for selecting studies (such as two independent reviewers) through each phase of the review (that is, screening, eligibility and inclusion in meta-analysis)
Data collection process	11c	Describe planned method of extracting data from reports (such as piloting forms, done independently, in duplicate), any processes for obtaining and confirming data from investigators
Data items	12	List and define all variables for which data will be sought (such as PICO items, funding sources), any pre-planned data assumptions and simplifications
Outcomes and prioritization	13	List and define all outcomes for which data will be sought, including prioritization of main and additional outcomes, with rationale
Risk of bias in individual studies	14	Describe anticipated methods for assessing risk of bias of individual studies, including whether this will be done at the outcome or study level, or both; state how this information will be used in data synthesis
Data synthesis	15a	Describe criteria under which study data will be quantitatively synthesised
	15b	If data are appropriate for quantitative synthesis, describe planned summary measures, methods of handling data and methods of combining data from studies, including any planned exploration of consistency (such as I^2, Kendall's τ)
	15c	Describe any proposed additional analyses (such as sensitivity or subgroup analyses, meta-regression)
	15d	If quantitative synthesis is not appropriate, describe the type of summary planned
Meta-bias(es)	16	Specify any planned assessment of meta-bias(es) (such as publication bias across studies, selective reporting within studies)
Confidence in cumulative evidence	17	Describe how the strength of the body of evidence will be assessed (such as GRADE)

*** It is strongly recommended that this checklist be read in conjunction with the PRISMA-P Explanation and Elaboration (cite when available) for important clarification on the items. Amendments to a review protocol should be tracked and dated. The copyright for PRISMA-P (including checklist) is held by the PRISMA-P Group and is distributed under a Creative Commons Attribution Licence 4.0.**

From: Shamseer L, Moher D, Clarke M, Ghersi D, Liberati A, Petticrew M, Shekelle P, Stewart L, PRISMA-P Group. Preferred reporting items for systematic review and meta-analysis protocols (PRISMA-P) 2015: elaboration and explanation. BMJ. 2015 Jan 2;349(jan02 1):g7647.

For peer review only

Research Checklist

PRISMA-P (Preferred Reporting Items for Systematic review and Meta-Analysis Protocols) 2015 checklist: recommended items to address in a systematic review protocol*

Section and topic	Item No	Checklist item	(Page No.#)
**ADMINISTRATIVE INFORMATION**			
Title:			
Identification	1a	Identify the report as a protocol of a systematic review	1
Update	1b	If the protocol is for an update of a previous systematic review, identify as such	Not applicable
Registration	2	If registered, provide the name of the registry (such as PROSPERO) and registration number	2
Authors:			
Contact	3a	Provide name, institutional affiliation, e-mail address of all protocol authors; provide physical mailing address of corresponding author	1
Contributions	3b	Describe contributions of protocol authors and identify the guarantor of the review	6
Amendments	4	If the protocol represents an amendment of a previously completed or published protocol, identify as such and list changes; otherwise, state plan for documenting important protocol amendments	Not applicable
Support:			
Sources	5a	Indicate sources of financial or other support for the review	6
Sponsor	5b	Provide name for the review funder and/or sponsor	
Role of sponsor or funder	5c	Describe roles of funder(s), sponsor(s), and/or institution(s), if any, in developing the protocol	
**INTRODUCTION**			
Rationale	6	Describe the rationale for the review in the context of what is already known	3
Objectives	7	Provide an explicit statement of the question(s) the review will address with reference to participants, interventions, comparators, and outcomes (PICO)	3
**METHODS**			
Eligibility criteria	8	Specify the study characteristics (such as PICO, study design, setting, time frame) and report characteristics (such as years considered, language, publication status) to be used as criteria for eligibility for the review	4

For peer review only

Information sources	9	Describe all intended information sources (such as electronic databases, contact with study authors, trial registers or other grey literature sources) with planned dates of coverage	5
Search strategy	10	Present draft of search strategy to be used for at least one electronic database, including planned limits, such that it could be repeated	5 (table 1)
Study records:			
Data management	11a	Describe the mechanism(s) that will be used to manage records and data throughout the review	5
Selection process	11b	State the process that will be used for selecting studies (such as two independent reviewers) through each phase of the review (that is, screening, eligibility and inclusion in meta-analysis)	5
Data collection process	11c	Describe planned method of extracting data from reports (such as piloting forms, done independently, in duplicate), any processes for obtaining and confirming data from investigators	6
Data items	12	List and define all variables for which data will be sought (such as PICO items, funding sources), any pre-planned data assumptions and simplifications	6
Outcomes and prioritization	13	List and define all outcomes for which data will be sought, including prioritization of main and additional outcomes, with rationale	4
Risk of bias in individual studies	14	Describe anticipated methods for assessing risk of bias of individual studies, including whether this will be done at the outcome or study level, or both; state how this information will be used in data synthesis	6
Data synthesis	15a	Describe criteria under which study data will be quantitatively synthesised	6
	15b	If data are appropriate for quantitative synthesis, describe planned summary measures, methods of handling data and methods of combining data from studies, including any planned exploration of consistency (such as I^2, Kendall's τ)	6
	15c	Describe any proposed additional analyses (such as sensitivity or subgroup analyses, meta-regression)	6
	15d	If quantitative synthesis is not appropriate, describe the type of summary planned	6
Meta-bias(es)	16	Specify any planned assessment of meta-bias(es) (such as publication bias across studies, selective reporting within studies)	6
Confidence in cumulative evidence	17	Describe how the strength of the body of evidence will be assessed (such as GRADE)	6

*** It is strongly recommended that this checklist be read in conjunction with the PRISMA-P Explanation and Elaboration (cite when available) for important clarification on the items. Amendments to a review protocol should be tracked and dated. The copyright for PRISMA-P (including checklist) is held by the PRISMA-P Group and is distributed under a Creative Commons Attribution Licence 4.0.**

From: Shamseer L, Moher D, Clarke M, Ghersi D, Liberati A, Petticrew M, Shekelle P, Stewart L, PRISMA-P Group. Preferred reporting items for systematic review and meta-analysis protocols (PRISMA-P) 2015: elaboration and explanation. BMJ. 2015 Jan 2;349(jan02 1):g7647.

For peer review only
